# EHMTI-0108. Efficacy of therapy in patients with migraine: comparison in group of < 9 migraines and > 10 migraines per month

**DOI:** 10.1186/1129-2377-15-S1-G40

**Published:** 2014-09-18

**Authors:** V Vukovic Cvetkovic, S Zupanic, M Bosnar Puretic

**Affiliations:** 1 Department of Neurology, Univesity Hospital Center "Sestre Milosrdnice", Zagreb, Croatia; 2 General Practice "Krunoslav Reljanovic, General Practice "Krunoslav Reljanovic, Zagreb, Croatia; 3 Department of Neurology, University Hospital Center Sestre milosrdnice, Zagreb, Croatia

## Aims

To investigate the efficacy of acute and prophylactic migraine therapy in groups of patients with < 9 migraines and > 10 migraines per month (p.m.).

## Methods

All consecutive patients with migraine that attended one single tertiary outpatient care service during 2011. were analysed. Migraine was diagnosed according to the ICHD II criteria.

## Results

A total of 380 patients were analysed; 262 (68.9%) patients with < 9 migraines pm (222 women, 40 men) (group A), 39 (10.3%) patients with 10-14 migraines p.m. (30 women, 9 men) (group B) and 79 (20.8%) patients with > 15 migraines p.m. (66 women, 13 men) (group C). In group A 72.1% declared that triptans were efficient, 8.1% partly and 12.6 not efficient; in group B 82.8% declared that triptans were efficient and 18.2 partly efficient; in group C 44.4% declared that triptans were very efficient, 22.2% partly and 33.3% not efficient. Adverse events were present in 9%, 9.1% and 11.1% respectively. In group A 102 (38.9%) was on prophylaxis, 47.1% declared efficacy > 50%, 18.6% declared efficacy 25-50% and 21.6% declared efficacy less than 25%. In group B 14 (35.9)% was on prophylaxis, 14.3% declared efficacy > 50%, 7.1% declared efficacy 25-50% and 57.1% declared efficacy less than 25%. In group C 24 (30.4%) was on prophylaxis, 4.2% declared efficacy > 50%, and 79.2% declared efficacy less than 25%. Adverse events were present in 9.3%, 21.4% and16.7% respectively.

## Conclusions

Migraine patients with less frequent attacks respond better to acute and prophylactic therapy. More attention should be given to patients with frequent attacks in order to provide them more efficient therapy.

No conflict of interest.

